# Genome-wide association analysis of stress tolerance indices in an interspecific population of chickpea

**DOI:** 10.3389/fpls.2022.933277

**Published:** 2022-08-19

**Authors:** Shweta Kalve, Krishna Kishore Gali, Bunyamin Tar’an

**Affiliations:** Department of Plant Sciences, University of Saskatchewan, Saskatoon, SK, Canada

**Keywords:** suboptimal conditions, interspecific crosses, marker-assisted introgression, wild chickpea, cultivars, stress tolerance indices

## Abstract

Chickpea is a cool season crop that is highly vulnerable to abiotic stresses such as heat and drought. High temperature during early flowering and pod development stages significantly reduces the crop yield. The wild relatives of chickpeas can be potential donors for the introgression of heat and drought tolerance into cultivated chickpeas for crop improvement. Initially, 600 interspecific lines were derived from crosses between two elite cultivars, CDC Leader (kabuli chickpea) and CDC Consul (desi chickpea), and 20 accessions of *Cicer reticulatum*. The F_5_ interspecific lines were tested for agronomic and seed quality traits including reaction to ascochyta blight disease under field conditions at two locations in 2018. A subset of 195 lines were selected based on resistance to ascochyta blight and acceptable seed quality. These lines were evaluated for their performance under suboptimal conditions at Lucky Lake (2019 and 2020) and Moose Jaw (2019), Saskatchewan, Canada, and Yuma, Arizona, United States (2019–2020). The lines were grown and evaluated at two seeding dates, normal (SD1) and late (SD2) seeding dates, at each location and year. The same lines were genotyped using Cicer60K Axiom® SNP chip. The population structure was determined based on 35,431 informative SNPs using fastStructure, and the interspecific lines were clustered at a *k*-value of 15. Significant marker-trait associations were identified for seed yield from SD1 and SD2 seeding dates, and stress tolerance indices (ATI, K_1_STI, MP, SSPI, and TOL) using phenotypic values both from individual locations and combined analyses based on BLUP values. SNP marker Ca2_34600347 was significantly associated with yield from both the seeding dates. This and other SNP markers identified in this study may be useful for marker-assisted introgression of abiotic stress tolerance in chickpea.

## Introduction

Chickpea (*Cicer arietinum* L.) is the world’s second most important pulse crop after the common bean (Varshney et al., 2013; Rani et al., 2020). It was one of the earliest domesticated legume crops and is currently grown in 59 countries. In 2019, the world production of chickpeas was around 14.2 million tons (FAO, 2019). Among abiotic stresses, drought and heat are the major environmental constraints limiting chickpea production worldwide in recent years (Devasirvatham and Tan, 2018; Rani et al., 2020). It has been reported that heat and drought can cause more than 70% yield loss in chickpea (Varshney et al., 2019). Chickpeas are mostly grown under rainfed conditions without irrigation. Therefore, soil moisture deficit toward the end of the crop season (terminal drought) affects about two-thirds of the global chickpea area (Gaur et al., 2008, 2019). Moreover, being a cool season food legume, chickpea yield is sensitive to heat stress exposure during the reproductive stage (Devasirvatham et al., 2015; Gaur et al., 2015).

Extreme heat and dry conditions are among the main abiotic stresses that affect crop yield across Canada. The prolonged heat wave and lack of precipitation in recent years in Western Canada^1^ has had an adverse impact on chickpea yield. Tolerance to abiotic stresses such as heat and drought is a complex trait that is the result of various morphological, physiological, and biochemical changes in plants (Kaloki et al., 2019). Moreover, these abiotic stresses are quantitatively inherited with a large effect of genotype x environment interaction (Jha et al., 2014). The development of cultivars with abiotic stress tolerance and yield stability is critical in chickpea breeding programs. However, very slow progress has been made in developing tolerant cultivars due to the physiological and genetic complexity of the trait. The variability and unpredictability of stress conditions during trials limit the selection efficiency. Therefore, knowledge of the traits responsible for the adaptation of chickpea to suboptimal environments is important for the development of cultivars with improved abiotic stress tolerance.

Crop wild relatives (CWR) preserve higher levels of genetic diversity as they have been challenged in natural environments for many years in comparison to domesticated cultivars. Hence, these wild relatives are crucial genetic resources used by plant breeders for crop improvement (Hajjar and Hodgkin, 2007). CWR has been used as a source of abiotic stress tolerance in many cultivated species (Hajjar and Hodgkin, 2007; Zhang et al., 2019). Recent advances in genotyping, breeding, and genomics have accelerated the use of CWR for crop improvement by marker-assisted introgression of wild alleles into cultivated germplasm (Bohra et al., 2021). Few examples included enhanced drought tolerance in cultivated germplasm of sunflower (Hussain et al., 2019), and improved drought related traits such as water use efficiency, earliness, and yield of cultivated groundnut by introgression of alleles from the wild groundnut species *Arachis duranensis* and *Arachis batizocoi* (Dutra et al., 2018). Another example is enhanced drought resistance and productivity of elite durum (*Triticum turgidum* ssp. *durum*) and bread wheat (*Triticum aestivum* L.) cultivars by the reintroduction of alleles from wild emmer wheat (Merchuk-Ovnat et al., 2017). More studies that have demonstrated wild progenitors as a valuable source for the enrichment of the domesticated gene pool for abiotic stress tolerance included reintroducing wild alleles in lentils (Gorim and Vandenberg, 2017) and wheat (Placido et al., 2013).

Genome-Wide Association Study (GWAS) provides a higher resolution of marker-trait association than classical QTL analysis using bi-parental populations (Korte and Farlow, 2013). Both GWAS and QTL mapping studies were used to identify the genetic loci associated with various abiotic stresses including drought and heat stress (Jha et al., 2021). Nested association mapping (NAM) has been a valuable approach to dissecting the genetic architecture of complex quantitative traits (Gangurde et al., 2020; Lakmes et al., 2022). NAM population consists of multiple families of recombinant inbred lines (RILs) derived from multiple inbred lines crossed to a single reference inbred line (Yu et al., 2008). It utilizes the combined power of QTL mapping and association mapping to identify the trait-associated markers (Buckler et al., 2009). A publicly available collection of wild chickpeas, especially *Cicer reticulatum*, which survives under suboptimal environment, is an important resource to improve stress tolerance in current chickpea cultivars (Singh et al., 2008; von Wettberg et al., 2018). Linkage drag is a known bottleneck for the introgression of QTLs from wild accessions to cultivated. To overcome the linkage drag and to introgress and expand the genetic basis of cultivated chickpea, introgression of wild alleles from multiple accessions of *C. reticulatum* into cultivated chickpea germplasm has been initiated at the University of Saskatchewan chickpea breeding program. The main objective of the current study was to examine the performance of the interspecific population derived from *C. arietinum* x *C. reticulatum* crosses under suboptimal conditions and to identify the genetic loci associated with the traits crucial for plant performance under suboptimal environments using genome-wide association analysis.

## Materials and methods

### Plant materials

A chickpea interspecific population consisting of 600 lines derived from crosses between elite cultivars (*C. arietinum*) and 20 accessions of *Cicer reticulatum* were developed. The elite cultivars were CDC Leader (kabuli chickpea) and CDC Consul (desi chickpea). The design of this population is like a nested association mapping (NAM) design. Each of the 20 *C. reticulatum* accessions (tester lines) was crossed with CDC Leader and CDC Consul (founder lines). A single F_1_ seed from each cross was grown in the greenhouse in 2014 and selfed to produce the F_2_ seed. The F_2_ plants of each cross were advanced to F_5_ as single seed descents (SSDs). The seeds of each F_5_ line were bulked and used for the evaluation of their agronomic performance including their reaction to ascochyta blight disease and seed visual quality at two locations, Limerick and Lucky Lake, SK during the 2018 growing season. One hundred and ninety-five F_7_ interspecific lines with improved resistance to ascochyta blight and acceptable visual seed quality were selected and used in the current study. All the selected lines were derived from crosses with CDC Leader as a common parent.

### Growing conditions and phenotypic data analysis

The 195 F_7_ interspecific lines were grown at Lucky Lake (2019 and 2020) and Moose Jaw (2019), Saskatchewan, Canada, and Yuma, Arizona, United States (2019–2020). The population was planted at two different seeding dates [normal (SD1) and late seeding (SD2)] at each location and year. The purpose of late seeding was to expose the plants to higher temperatures during flowering. The normal seeding date in Saskatchewan was in the second week of May (14 May in Moose Jaw, 2019, 17 May in Lucky Lake, 2019, and 12 May in Lucky Lake, 2020) while the late seeding was planted around 2 weeks after the normal seeding (28 May in all the locations; Supplementary Table 1). At Yuma, Arizona, the first seeding was on 6 November while the second seeding was on 14 January (Supplementary Table 1) to maximize the chance that the population was exposed to a temperature above 27°C during flowering. At each location, the lines were planted in a one square meter plot. In each plot, the seeds were planted in three rows with a density of 60 seeds m^−2^. A randomized complete block design (RCBD) with three replications was used at each location and seeding date. Daily maximum and minimum temperatures and other meteorological data were recorded at each location. There were multiple occurrences of temperatures above 26°C–27°C in all the locations during flowering on late seeding dates (Supplementary Table 1).

Days to flowering (DTF) were recorded for each line on the plot basis when 50% of the plants had flowered. Days to maturity (DTM) was also documented for each line when 50% of the plants in each plot were matured. Plant height was measured from the ground level to the tip of the plants when the pods reached physiological maturity. Flower color was recorded based on visual observation during flowering. Seed weight was measured by weighing 1,000 seeds per line after harvest. Reaction to ascochyta blight of each line was recorded on a plot basis, using a mixed quantitative and qualitative 0–9 score scale as described by Chongo et al. (2004). Seed yield was measured on a plot basis. Seed yield under stressed (late seeding) and non-stressed (normal seeding) conditions were used to predict the stress tolerance index of each line. The different stress indices used to assess stress tolerance included tolerance index (TOL), mean productivity (MP), abiotic tolerance index (ATI), stress susceptibility percentage index (SSPI), and modified stress tolerance index (K_1_STI). These have been reported as reproducible indices under severe stress conditions in chickpeas (Farshadfar and Geravandi, 2013). The following formulas were used to calculate the stress indices.

TOL = Yp–Ys (Rosielle and Hamblin, 1981).
MP = (Yp + Ys)/2 (Rosielle and Hamblin, 1981).
ATI = [(Yp–Ys)/(Y̅p/Y̅s)] × 100 (Moosavi et al., 2008).
SSPI = [(Yp–Ys)/(2Y̅p)] × 100 (Moosavi et al., 2008).
K_1_STI = (Yp2/Y̅p2) × [(Yp + Ys)/Y̅p2)] (Farshadfar and Sutka, 2003).

In the above formulas, Ys, Yp, Y̅s, and Y̅p represent seed yield in stress and non-stress conditions for each genotype, and mean seed yield in stress and non-stress conditions for all genotypes, respectively.

For field evaluation, both combined analyses across locations and years for each seeding date, and separate analyses on each year (2019 and 2020) for each seeding date were conducted. ANOVA was done using PROC MIXED in which genotypes were considered as a fixed factor and years as a random factor. The LSMEANS statement was used to compute the average phenotypic score for each line. For a separate analysis of each year, ANOVA was done using the PROC MIXED procedure, in which the lines were considered as a fixed factor and replication was considered as a random factor. To estimate the broad sense heritability (H^2^), variance components were calculated using the SAS PROC VARCOMP procedure (SAS Institute Inc, 1999). The H^2^ of various phenotypic responses at plot level based on individual experiments and over the years were estimated using the following two equations, respectively


$$H^{2}=\frac{\sigma^{2}G}{\sigma^{2}G+\sigma^{2}er}\;\mathrm{and}\;H^{2}=\frac{\sigma^{2}G}{\sigma^{2}G+\sigma^{2}GY+\sigma er}$$

where σ^2^G, σ^2^Y, σ^2^GY, and σ^2^er are estimates of genotype, site-year, genotype by site-year interaction, and error variance, respectively (Singh et al., 1993). Spearmen’s rank correlation coefficients between the seed yields obtained under stress and non-stress conditions and the tolerance indices for each site year were calculated.

### Genotyping of mapping population

The interspecific population was genotyped using a 60 K Axiom® SNP array (61,335 SNPs) at Eurofins, WI, United States. Individual plants from each line were grown under controlled conditions in phytotron chambers. Young leaf tissue was harvested from 2 to 3 weeks old plants. These leaf tissues were freeze dried using Labconco FreeZone 6-L Console Freeze Dry System and sent to Eurofins BioDiagnostics, WI, United States, for DNA extraction and genotyping.

### Population structure and linkage disequilibrium analysis

The program fastSTRUCTURE (Raj et al., 2014) was used to estimate the most likely number of clusters (*K*) into which the interspecific population can be grouped, and their degree of admixtures, based on genotypic data of 35,431 SNPs. The value of *K* was determined based on the lowest prediction error, and the smallest number of iterations for convergence. For each line, the value of *Q,* which is the probability of belonging to one of the clusters was derived from the matrix of contributions. A shared allele index derived from the dissimilarity matrix estimated from the SNP genotypic data were used to construct an unweighted neighbor-joining tree (Perrier et al., 2003).

Linkage disequilibrium (LD) of each chromosome was calculated as the correlation between marker-pairs among the SNP markers of each chromosome. The calculated Pearson correlation coefficient [r] was used to calculate LD decay by Quantile regression (R package “quantreg”; Koenker, 2017). The LD decay was calculated by plotting r^2^ values as a function of genetic distance.

### Association mapping

The software program GAPIT (Genome Association and Prediction Integrated Tool—R package: Lipka et al., 2012) was used to determine the association between SNP genotypes and the tolerance indices of the population. The phenotypes measured in individual environments were used for association analysis. Simultaneously, the best linear unbiased predictors (BLUP) of each trait of four environments tested were calculated using the “Ime4” package of the R3.6.1 software,^2^ considering environmental effects as fixed and genotype as random: y ∼ (1 |rep% in % env) + (1|env) + (1 |lines) + (1 |env: lines), where rep% in % env represents replications were nested within the environments. The formula for the best linear unbiased estimate (BLUP) of phenotype is: y = Xb + Zu + e, where y, b, u, and e represent the observed phenotype, fixed effect vector, random effect vector, and residual, respectively, and X and Z represent incidence matrices. For the association analysis, the kinship coefficient matrix (K) values calculated by GAPIT by identity-by-state (IBS), were used. Multiple models of the GAPIT program including mixed linear model (MLM), multi-linear mixed model (MLMM), compressed mixed linear model (CMLM), general linear model (GLM), settlement of mixed linear models under progressively exclusive relationship (SUPER), fixed and random model circulating probability unification (FarmCPU) and factored spectrally transformed linear mixed models (FaST-LMM’; Tang et al., 2016; Kaler et al., 2020) were tested for comparison of the association analysis (Supplementary Figure 1). By comparison of the Q–Q plots of each model, which are drawn by plotting the observed and expected log_10_ *p*-values, the MLMM model was chosen to report marker-trait associations.

## Results

### Phenotypic evaluation

Various phenotypic traits were recorded across the locations and seeding dates in 2019 and 2020 (Tables 1A,B). ANOVA showed significant differences among the interspecific lines for days to flowering, days to maturity, seed yield, flower color, plant height, seed size, and ascochyta blight severity across all locations for normal (SD1) and late seeding (SD2) treatments in 2019 and 2020. There were significant effects of the environment (year) and genotype by environment (year) interaction in all the phenotypic traits recorded for both seeding dates. The interaction of genotype and site year was not significant for plant height in SD2 (Table 1B). Some of the phenotypic traits were not recorded in all four field trials because of a lack of resources and the restrictions due to COVID-19, especially during the 2019–2020 trials at Yuma, AZ. Plot yield was among the phenotypic traits measured in all years, locations, and seeding dates. Moreover, in previous studies seed yield was found to be an important trait to measure tolerance under stress and non-stress environments. The seed yield data were used for the calculation of stress tolerance indices and association analysis. The mean value and range of population for each trait are presented in Table 2.

**Table 1:** ANOVA for days to flowering (DTF), days to maturity (DTM), seed yield, flower color, plant height, seed size (1,000 seed weight), and ascochyta blight rating under (A) normal seeding (SD1) and (B) late seeding (SD2) for the interspecific population was evaluated under field conditions at Lucky Lake and Moose Jaw, Saskatchewan in 2019, Yuma, Arizona in 2019–2020, and Lucky Lake, Saskatchewan in 2020.

Year/locations	Effect	*F* value						
		DTF	DTM	Seed yield (g)	Flower color	Plant height (cm)	Seed size (g)	Ascochyta blight rating
**(A)**								
Field combined years (2019 and 2020)	G	7.36^***^	3.38^***^	5.49^***^	12.08^***^	2.82^***^	25.86^***^	Only 1 year data available
	Y	404.08^***^	252.36^***^	237.41^***^	1.15^***^	179.43^***^	1115.76^***^	
	G*Y	1.27^***^	3.41^***^	2.01^***^	3.24^***^	1.51^***^	3.29^***^	
	σ^2^G	3.79	−0.32	3887.6	0.19	10.06	897.73	
	σ^2^Y	3.9	6.79	5470.2	0.02	13.9	910.83	
	σ^2^GY	0.59	12.08	4714.3	0.1	8.66	348.55	
	σ^2^er	5.5	14.51	12864.9	0.12	46.81	465.58	
	H^2^	0.38	−0.01	0.18	0.46	0.15	0.52	
**Individual year/location**								
Lucky Lake, 2019	G	7.32^***^	3.12^***^	3.27^***^	7.6^***^	2.07^***^	11.93^***^	No symptoms
	σ^2^G	2.46	5.59	3860.8	0.23	15.31	1685.3	
	σ^2^er	1.23	7.62	5095.7	0.1	41.87	452.63	
	H^2^	0.67	0.42	0.43	0.7	0.27	0.79	
Moose Jaw, 2019	G	2.85^***^	NA	2.31^***^	NA	NA	13.56^***^	NA
	σ^2^G	5.35	NA	2588.3	NA	NA	1663.8	NA
	σ^2^er	8.26	NA	5994.7	NA	NA	376.24	NA
	H^2^	0.39	NA	0.3	NA	NA	0.82	NA
Yuma, 2019–-2020	G	NA	NA	2.69^***^	NA	NA	6.39^***^	NA
	σ^2^G	NA	NA	23901.2	NA	NA	672.38	NA
	σ^2^er	NA	NA	36115.4	NA	NA	423.19	NA
	H^2^	NA	NA	0.4	NA	NA	0.61	NA
Lucky Lake, 2020	G	3.24^***^	3.53^***^	4.4^***^	7.9^***^	2.3^***^	5.57^***^	1.81^***^
	σ^2^G	5.24	17.31	5346.4	0.33	22.23	932.14	0.36
	σ^2^er	7.01	21.87	4060.6	0.15	51.42	644.15	1.33
	H^2^	0.43	0.44	0.57	0.7	0.3	0.59	0.21
**(B)**								
Field combined years (2019 and 2020)	G	1.92^***^	2.15^***^	6.5^***^	15.05^***^	3.32^***^	16.3^***^	1.84^***^
	Y	66.36^***^	2540.84^***^	355.52^***^	6.83^**^	6.96^**^	131.91^***^	4574.95^***^
	G*Y	1.21^*^	1.35^**^	1.77^***^	2.04^***^	0.93^ns^	4.15^***^	1.42^**^
	σ^2^G	1.98	3.27	1929.5	0.31	13.93	825.95	0.001
	σ^2^Y	1.95	91.04	2,796	0.0009	0.38	91.82	3.57
	σ^2^GY	0.98	2.06	1242.6	0.05	−0.44	421.05	0.09
	σ^2^er	17.28	20.18	3970.8	0.14	34.77	388.51	0.47
	H^2^	0.1	0.13	0.27	0.61	0.29	0.51	0.002
**Individual year/location**								
Lucky Lake, 2019	G	1.29^*^	1.23^*^	2.32^***^	7.19^***^	2.17^***^	NA	1.35^**^
	σ^2^G	3.02	1.37	2341.8	0.33	13.27	NA	0.03
	σ^2^er	32.57	27.7	5111.5	0.16	33.62	NA	0.36
	H^2^	0.08	0.05	0.32	0.67	0.28	NA	0.07
Moose Jaw, 2019	G	NA	NA	2.81^***^	NA	NA	NA	NA
	σ^2^G	NA	NA	3026.2	NA	NA	NA	NA
	σ^2^er	NA	NA	4649.5	NA	NA	NA	NA
	H^2^	NA	NA	0.39	NA	NA	NA	NA
Yuma, 2019–2020	G	NA	NA	2.39^***^	NA	NA	8.2^***^	NA
	σ^2^G	NA	NA	1335.4	NA	NA	1340.5	NA
	σ^2^er	NA	NA	2411.9	NA	NA	513.19	NA
	H^2^	NA	NA	0.36	NA	NA	0.72	NA
Lucky Lake, 2020	G	4.55^***^	2.74^***^	4.97^***^	10.39^***^	2.14^***^	14.04^***^	1.87^***^
	σ^2^G	2.9	8.1	5139.7	0.38	13.67	1158.2	0.16
	σ^2^er	2.45	13.94	3798.7	0.12	35.89	269.43	0.56
	H^2^	0.54	0.37	0.58	0.76	0.27	0.81	0.22

G, genotype interaction; Y, site year interaction; G*Y, genotype by site year interaction; σ^2^G, σ^2^Y, σ^2^GY, and σ^2^er are estimates of genotype, site year, genotype by site year interaction, and error variance, respectively; H^2^ is broad sense heritability; ns, not significant; NA, data not available.

*** Indicates a significant difference at *P* ≤ 0.001.

** Indicates a significant difference at *P* ≤ 0.01.

* Indicates a significant difference at *P* ≤ 0.05.

**Table 2:** Mean and range values of the interspecific population for characters assessed under field conditions.

		Population			
		SD1		SD2	
Character	Location	Mean	Range	Mean	Range
Days to flowering	Lucky Lake, 2019	50.75	45–56	49.57	42–60
	Moose Jaw, 2019	NA	NA	NA	NA
	Yuma 2019–2020	NA	NA	NA	NA
	Lucky Lake 2020	54.28	48–64	47.59	42–56
Days to maturity	Lucky Lake, 2019	98.88	94–108	102.61	89–110
	Moose Jaw, 2019	NA	NA	NA	NA
	Yuma 2019–2020	NA	NA	NA	NA
	Lucky Lake 2020	102.37	90–118	89.23	79–97
Seed yield (g)	Lucky Lake, 2019	227.97	28–443	192.62	22–395
	Moose Jaw, 2019	306.6	90–486	210.14	43–385
	Yuma 2019–2020	259.24	5–1,638	89.54	3–282
	Lucky Lake 2020	128.85	6–466	146.67	9–467
Plant height (cm)	Lucky Lake, 2019	42.67	29–61	44.99	35–67
	Moose Jaw, 2019	NA	NA	NA	NA
	Yuma 2019–-2020	NA	NA	NA	NA
	Lucky Lake 2020	47.81	27–66	45.7	31–63
Seed size (1,000 seed weight in g)	Lucky Lake, 2019	172.88	77–343	NA	NA
	Moose Jaw, 2019	202.54	111–330	NA	NA
	Yuma 2019–2020	131.09	48–261	165.67	55–316
	Lucky Lake 2020	165.86	61–267	177.67	87–303
Ascochyta blight rating	Lucky Lake, 2019	No symptoms	No symptoms	5.27	4.5–7.5
	Moose Jaw, 2019	NA	NA	NA	NA
	Yuma 2019–2020	NA	NA	NA	NA
	Lucky Lake 2020	0.43	0–4	2.59	2–5

### Correlation of tolerance indices under field conditions

Late seeding (stress condition) lowered the yield in comparison to normal seeding (non-stress condition) in all site years, except Lucky Lake in 2020 where the difference in seed yield was marginal but significant (*p* < 0.02; Table 2). Spearman’s rank correlation coefficients between the seed yields obtained under normal and late seeding and the stress tolerance indices for each year were calculated (Table 3). The correlation between the seed yield ranks under normal (non-stress) and late seeding (stress) conditions were positive and significant (*p* ≤ 0.001) for all site years, indicating a change in the ranking of the genotypes for seed yield production due to stress.

**Table 3:** Spearmen’s rank correlation between different stress tolerance indices for each site years.

Year/location	Parameters	Yp	Ys	TOL	MP	ATI	SSPI
Lucky Lake, 2019	Ys	0.39^***^					
	TOL	0.58^***^	−0.46^***^				
	MP	0.83^***^	0.81^***^	0.09^*^			
	ATI	0.58^***^	−0.46^***^	1^***^	0.09^*^		
	SSPI	0.58^***^	−0.46^***^	1^***^	0.09^*^	1^***^	
	K_1_STI	0.98^***^	0.52^***^	0.45^***^	0.91^***^	0.45^***^	0.45^***^
Moose Jaw, 2019	Ys	0.36^***^					
	TOL	0.58^***^	−0.51^***^				
	MP	0.82^***^	0.80^***^	0.06^ns^			
	ATI	0.58^***^	−0.51^***^	1^***^	0.06^ns^		
	SSPI	0.58^***^	−0.51^***^	1^***^	0.06^ns^	1^***^	
	K_1_STI	0.98^***^	0.50^***^	0.43^***^	0.91^***^	0.43^***^	0.43^***^
Yuma 2019–2020	Ys	0.31^***^					
	TOL	0.91^***^	−0.04^ns^				
	MP	0.95^***^	0.55^***^	0.76^***^			
	ATI	0.91^***^	−0.04^ns^	1^***^	0.76^***^		
	SSPI	0.91^***^	−0.04^ns^	1^***^	0.76^***^	1^***^	
	K_1_STI	0.97^***^	0.33^***^	0.89^***^	0.94^***^	0.89^***^	0.89^***^
Lucky Lake 2020	Ys	0.55^***^					
	TOL	0.41^***^	−0.48^***^				
	MP	0.85^***^	0.89^***^	−0.06^ns^			
	ATI	0.41^***^	−0.48^***^	1^***^	−0.06^ns^		
	SSPI	0.41^***^	−0.48^***^	1^***^	−0.06^ns^	1^***^	
	K_1_STI	0.99^***^	0.65^***^	0.29^***^	0.91^***^	0.29^***^	0.29^***^

Yp, seed yield under non-stress; Ys, seed yield under stress; TOL, tolerance index; MP, mean productivity; ATI, abiotic tolerance index; SSPI, stress susceptibility percentage index; K_1_STI, modified stress tolerance index.

* Indicates significance at *p* ≤ 0.05.

*** Indicates significance at *p* ≤ 0.001.

ns not significant.

Positive significant relationships were observed between the seed yield under non-stress conditions (Yp) and TOL, MP, ATI, SSPI, and K_1_STI in all site years. On the other hand, the seed yield under stress conditions (Ys) was positively correlated with MP and K_1_STI, but negatively correlated with TOL, ATI, and SSPI in all site years.

### Genotyping

A total of 35,429 SNPs were used to determine the population structure. Of these SNPs, 32,228 were located on the eight chromosomes of chickpea and 3,101 SNPs were on scaffolds and are not located on any of the chromosomes. After filtering for a minimum allele frequency (MAF) of 0.05, 20,679 SNPs were used for association analysis.

### Linkage disequilibrium analysis

**Figure 1:**
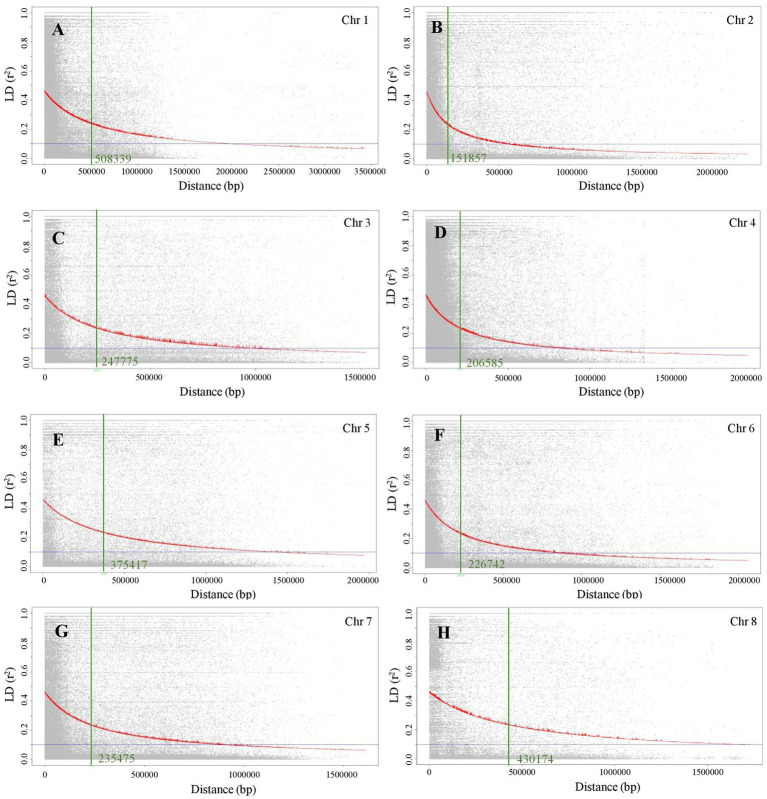
Linkage disequilibrium (LD) decay plots of eight chromosomes of chickpea. **(A)** Chromosome 1, **(B)** Chromosome 2, **(C)** Chromosome 3, **(D)** Chromosome 4, **(E)** Chromosome 5, **(F)** Chromosome 6, **(G)** Chromosome 7, and **(H)** Chromosome 8.

LD decay was calculated as the Pearson correlation coefficient (r^2^) between marker pairs of each chromosome. The LD decay differed among the eight chromosomes. The r^2^ _max,90,_ which is calculated as the maximum r^2^ in the 90th percentile of each chromosome, for chromosomes 1 to 8 is between 0.24 and 0.26. The physical distance in Mb at which LD of each chromosome has decayed to half of r^2^_max,90_ is 0.51, 0.15, 0.25, 0.21, 0.38, 0.23, 0.24, and 0.43 Mb, for chromosomes 1 to 8, respectively (Figure 1).

### Population genetic structure

**Figure 2:**
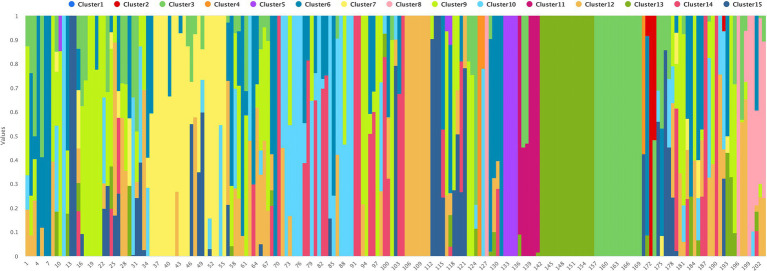
The population structure of 203 chickpea interspecific lines and accessions based on *k* = 15.

**Figure 3:**
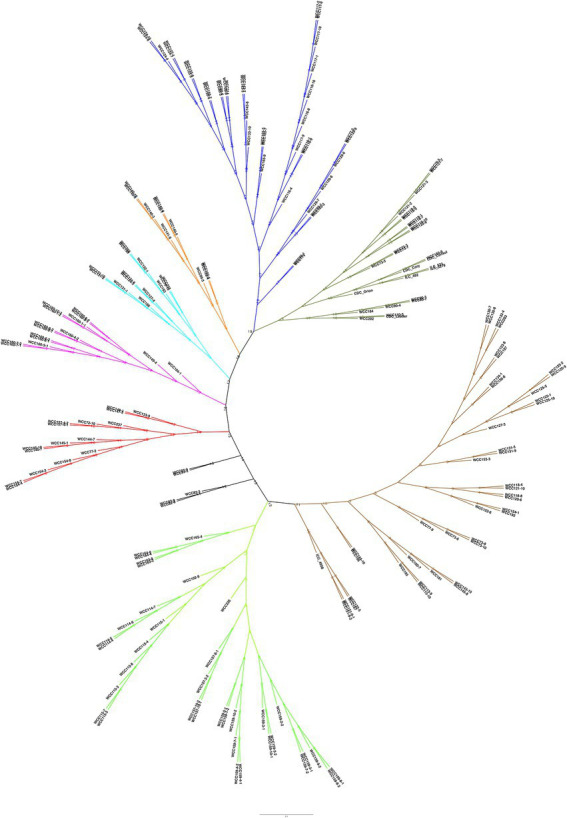
Genetic relatedness among the 203 chickpea accessions, estimated by neighbor-joining method.

To determine the population structure, the most likely number of clusters (*k*) was tested from 2 to 20. A *k*-value of 15 is best suited to describe the genetic structure of 195 chickpea interspecific lines along with the two parents CDC Consul and CDC Leader, and six CDC cultivars used as checks, CDC Orion, CDC Cory, ILC 533, ICC 4958, and ILC 3279. The probability of membership of individual lines in each cluster was estimated by admixture analysis (Figure 2; Supplementary Table 2). The grouping of the interspecific population into phylogenetic clusters based on the neighbor-joining (NJ) tree differed from the population structure analysis that the population is divided into eight clades (Figure 3).

### Association mapping

**Figure 4:**
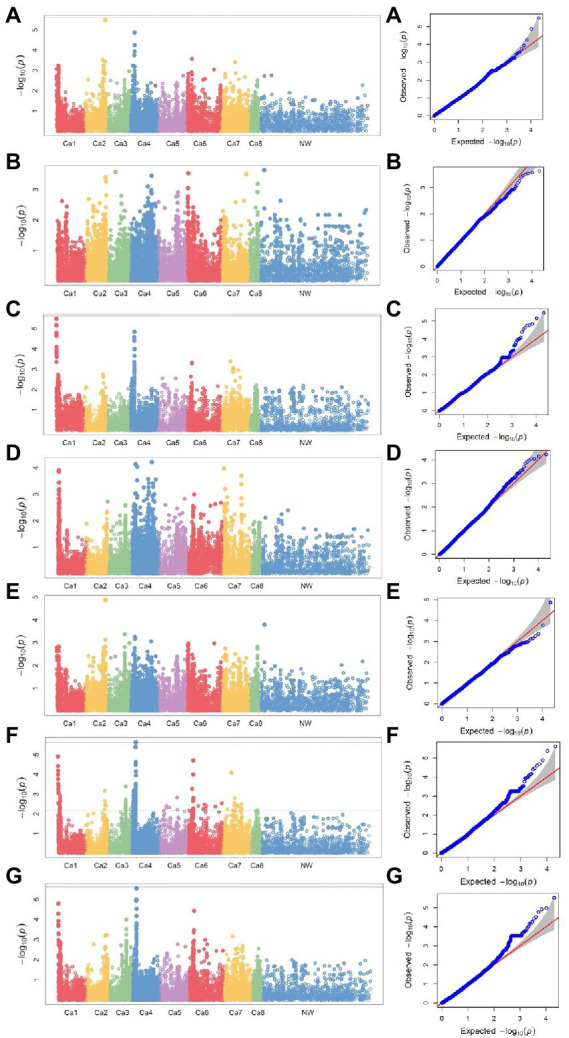
Manhattan plots of −log10 *p-*values and the corresponding quantile–quantile (Q–Q) plots of the association analysis for **(A)** seed yield under non-stress and **(B)** seed yield under stress conditions and yield indices **(C)** ATI, **(D)** K_1_STI, **(E)** MP, **(F)** SSPI, and **(G)** TOL using a mixed linear model for four locations: Lucky Lake and Moose Jaw, Saskatchewan, 2019, Yuma, Arizona, 2019–2020, and Lucky Lake, Saskatchewan, 2020. For Manhattan plots: *y*-axis, −log10 *p*-values; *x*-axis, chromosome numbers. For Q–Q plots: *y*-axis, observed −log10 *p*-values; *x*-axis, expected −log10 *p*-values.

The seed yield of interspecific lines measured both in normal and heat stress treatments, and the heat stress indices calculated based on the seed yield in both the treatments, were used to identify the associated SNP markers. The BLUP values calculated based on the measurements of each trait/index in four station-years were used for association analysis. SNP markers were identified for association with seed yield both in normal and heat stress environments, and the indices calculated based on the seed yield in these two treatments are listed in Table 4. SNP marker Ca2_34600347 was identified for association with grain yield both in normal and stress (late seeding) treatments. The markers for ATI and TOL are located on chromosome 1, while the markers for other indices K_1_STI and SSPI are located on chromosome 4. The Manhattan plots representing the genome-wide marker-trait associations, concerning the seven traits/indices measured, and their corresponding Q–Q plots are presented in Figure 4.

**Table 4:** Selected marker-trait associations, identified based on BLUP values of phenotypes measured in four station-years during 2019 and 2020.

Phenotypic trait/index	SNP marker	*P*-value	maf
Ys (seed yield under stress conditions)	NW_9270594	2.31E^−04^	0.31
	Ca3_15304269	2.61E^−04^	0.09
	Ca6_3396299	2.89E^−04^	0.31
	Ca7_43614232	3.14E^−04^	0.41
	Ca4_37419513	3.47E^−05^	0.40
	Ca2_34600347	3.92E^−04^	0.07
Yp (seed yield under non-stress conditions)	Ca2_34600347	3.25E^−06^	0.07
	Ca4_8694304	1.35E^−05^	0.05
	Ca4_8737135	5.55E^−05^	0.14
ATI (abiotic tolerance index)	Ca1_47259	3.42E^−06^	0.46
	Ca1_56428	6.91E^−06^	0.46
K_1_STI (modified stress tolerance index)	Ca4_36637574	6.05E^−05^	0.10
	Ca4_8646741	7.12E^−05^	0.16
	Ca4_11276937	9.00E^−05^	0.07
	Ca4_11277513	9.00E^−05^	0.07
MP (mean productivity)	Ca2_34600347	1.34E^−05^	0.07
SSPI (stress susceptibility percentage index)	Ca4_8694304	2.37E^−06^	0.05
	Ca4_8313845	4.17E^−06^	0.06
TOL (tolerance index)	Ca4_8694304	2.87E^−06^	0.05
	Ca4_8670257	1.04E^−05^	0.06
	Ca4_8313845	1.19E^−05^	0.06
	Ca1_47259	1.66E^−05^	0.46

maf, minimum allele frequency.

## Discussion

The wild relatives of chickpea have been an invaluable source for improving elite chickpea germplasm through resistance to biotic stress (Sharma et al., 2006; Singh et al., 2008). Moreover, crop wild relatives were reported to be an important resource for genetic improvement for various abiotic stresses (Singh et al., 2008; Coyne et al., 2020; Bakir et al., 2021). Collection of various wild chickpeas, mainly *C. reticulatum* was reported to have biotic and abiotic stress tolerance (Singh et al., 2008; von Wettberg et al., 2018; Bakir et al., 2021). *C. reticulatum* is in the primary gene pool of chickpea (Bakir et al., 2021). It is known for its crossing-compatibility with domesticated chickpea (*C. arietinum*) and generally produces fertile progeny because of good chromosome pairing (Bakir et al., 2021). Therefore, in the current study, we have used *C. reticulatum* to introgress stress tolerance in cultivated kabuli and desi chickpea (*C. arietinum*) germplasm. In this study, we developed progeny from interspecific crosses between adapted elite cultivars, CDC Leader and CDC Consul, and 20 *C. reticulatum* accessions. Further lines with improved response to ascochyta blight and acceptable seed quality were selected and used in this study. Interestingly, all these selected lines were derived from CDC Leader (kabuli chickpea) and 17 wild accessions. The design of our population is the same as the nested association mapping (NAM) design. Generally, the bi-parental population has a lack of mapping precision and low genetic diversity. Therefore, using NAM helps to capture additional genetic diversity and increase genetic recombination.

Chickpea is a dry season food legume that is mostly grown on residual moisture after the rainy season. Toward the end of the growing season chickpeas often experience terminal drought stress. Moreover, if the sowing is delayed, the crop may deal with heat stress during the reproductive phase (Gaur et al., 2019; Maphosa et al., 2020; Rani et al., 2020). Low moisture and high temperatures during the flowering and early pod filling stage can substantially influence chickpea yield by forcing early maturity resulting in low biomass and a low number of pods and seeds per plant (Maphosa et al., 2020; Rani et al., 2020). Therefore, to understand the performance of interspecific populations under a suboptimal environment, the population in this study was planted at two different seeding dates (normal and late seeding). The main objective of late seeding was to expose the plants to stress conditions such as higher temperatures and low moisture during flowering. Previous studies have also used the same approach to understand the effect of stress on crop development and yield in canary seeds (Miller, 1999). Furthermore, it was observed that late seeding has a negative effect on chickpea yield (Machado et al., 2006; McKenzie et al., 2006).

The current study evaluated various phenotypic traits such as days to flowering, days to maturity, seed yield, flower color, plant height, seed size, and ascochyta blight disease resistance on both the seeding dates. There were significant effects of environment and genotype by environment interaction in all the phenotypic traits documented for both seeding dates which clearly shows that a suboptimal environment negatively impacts crop development in chickpea. Similarly, other studies have shown the negative effect of stress on various phenotypic traits grown under stress and non-stress environments (Arif et al., 2021; Jeffrey et al., 2021; Jha et al., 2021). Yield is a crucial trait and an important indicator to define tolerance between stress and non-stress conditions and has been used to describe the performance of any genotype while screening in various environments (Kaloki et al., 2019; Pour-Aboughadareh et al., 2019). Moreover, in the current study seed yield was among the phenotypic traits measured in all years, locations, and seeding dates. Therefore, we have used seed yield to calculate various stress tolerance indices. Furthermore, crop yield under stress and non-stress conditions and stress tolerance indices were used for association analysis. Seed yield was significantly reduced in the late seeded population in all the locations as compared to the normal seeded population except Lucky Lake, 2020 which could be because of less stressed conditions while the late seeded population in Lucky Lake. Similar to our findings, previous studies have also shown reduced yield under late seeded populations (Machado et al., 2006; McKenzie et al., 2006). The calculation of spearman’s rank correlation coefficients between seed yields acquired under normal and late seeding and the stress tolerance indices for each year was done. We found that the correlation between seed yield under stress and non-stress conditions was positive and significant which shows that the yield production was different in all the genotypes under non-stress conditions. Similar results were also found in previous studies (Moosavi et al., 2008; Farshadfar and Geravandi, 2013). Moreover, seed yield under non-stress conditions was positively correlated with TOL, MP, ATI, SSPI, and K_1_STI which shows that selection of the genotypes based on these indices will improve the yield under non-stress conditions. Some studies have also found a positive correlation between non-stress conditions and MP, K_1_STI (Farshadfar and Geravandi, 2013) and TOL, MP, SSPI, ATI (Moosavi et al., 2008). On the other hand, the seed yield under stress conditions was positively correlated with MP and K_1_STI but negatively correlated with TOL, ATI, and SSPI indicating that the selection based on a higher value of MP and K_1_STI will improve seed yield while selection based on TOL, ATI, and SSPI will reduce seed yield under stress conditions. Therefore, correlation analysis between seed yield and stress tolerance indices can be a good criterion for screening the best genotypes and indices used.

Linkage drag is one of the major bottlenecks to using wild species in the genetic improvement of crop plants including chickpea. Often, a large portion of entire chromosomes is affected by the linkage drag, which is a hindrance to the introgression of desirable alleles. In the current study, the LD decay observed in each chromosome is from 0.15 to 0.51 Mb, indicating that the interspecific population used in the current study is suitable for the objectives of this study. Genome-wide association study (GWAS) was used to understand the genetic basis of complex traits in chickpea (Kohli et al., 2020) and heat stress in other legumes (Tafesse et al., 2020, 2021). Since we intend to identify the heat stress loci in wild chickpea, and as well to introgess the same in an elite chickpea genetic background, we developed an inbred line population derived from crosses of wild accessions and elite chickpea cultivars. The 195 inbred line population used for association mapping of heat stress tolerance was an interspecific population derived from crosses of CDC Leader with 17 *C. reticulatum* accessions.

In the current study, we used the measurement of seed yield in heat stress conditions as an important criterion to identify heat stress tolerance in chickpea interspecific lines. Understanding the genetic basis of yield in heat stress conditions is important to develop heat stress tolerant high yielding chickpea varieties. Previous studies have identified quantitative trait loci (QTLs) associated with yield (Barmukh et al., 2021), outlining component traits and heat tolerance (Thudi et al., 2014) in chickpea. Barmukh et al. (2021) identified yield-associated QTLs on LG1 and LG4 using a bi-parental mapping population of ICC 4958 × DCP 92–3. In the current study, we identified markers associated with seed yield and heat tolerance indices mainly on LG1 and LG4, while markers associated with seed yield in heat stress were distributed on multiple linkage groups including LG4. Yield is a complex trait and is determined by several components such as pod weight, pod number, and the number of reproductive nodes, etc. Tafesse et al. (2020, 2021) identified the correlation between multiple components of heat stress and SNP markers associated with these components in a GWAS study on peas. We have used the measurement of seed yield in heat stress conditions as a key indicator of heat tolerance, and the indices calculated based on seed yield in normal and heat stress conditions were among the other indicators used. The SNP marker Ca2_34600347, which was identified for its association with seed yield both in normal and heat stress environments, could be a valuable marker for the marker-assisted selection of heat tolerance in chickpea.

## Conclusion

Wild relatives are a source of novel alleles to improve chickpea adaptation to suboptimal environments. In the current study, we used an interspecific population derived from *C. reticulatum* accessions. The late seeding and exposure of this population to suboptimal growth conditions has significantly reduced the seed yield. The study characterized the variation of the interspecific population for its yield performance and yield-related indices under suboptimal conditions. Individual lines that were identified for superior performance in suboptimal conditions can be used as trait donors in breeding for stress tolerance. The trait-associated SNP markers identified can be used for marker-assisted selection in the breeding pipeline. Future detailed analysis of these SNPs would allow us to identify the genes underlying tolerance to abiotic stress, providing new alleles and molecular markers to use in chickpea crop improvement.

## Data availability statement

The original contributions presented in the study are included in the article/Supplementary material, further inquiries can be directed to the corresponding author.

## Author contributions

SK conducted all the experiments, analyzed phenotypic data, and wrote the manuscript. KG analyzed genotypic data, conducted association analysis, and wrote the manuscript. BT conceived, designed, and supervised the research and reviewed the manuscript. All authors contributed to the article and approved the submitted version.

## Funding

We acknowledge the financial support from the Saskatchewan Ministry of Agriculture through the Agricultural Development Fund.

## Conflict of interest

The authors declare that the research was conducted in the absence of any commercial or financial relationships that could be construed as a potential conflict of interest.

## Publisher’s note

All claims expressed in this article are solely those of the authors and do not necessarily represent those of their affiliated organizations, or those of the publisher, the editors and the reviewers. Any product that may be evaluated in this article, or claim that may be made by its manufacturer, is not guaranteed or endorsed by the publisher.

## Footnotes

^1^ https://www.canada.ca/en/environment-climate-change/news/2021/06/environment-and-climate-change-canada-warns-western-canadians-about-dangerous-record-high-temperatures.html

^2^ www.r-project.org

## Supplementary material

SUPPLEMENTARY FIGURE 1 Q–Q plots of the association analysis for seed yield under non-stress (Yp), seed yield under stress conditions (Ys) and yield indices ATI, K1STI, MP, SSPI, and TOL from various GWAS models (CMLM, FarmCPU, FaST-LMM, GLM, MLM, MLMM, SUPER).

SUPPLEMENTARY TABLE 1 Daily temperature and precipitation recorded at Lucky Lake and Moose Jaw, Saskatchewan, Canada in 2019, Yuma, Arizona, USA in 2019–2020 and Lucky Lake, Saskatchewan, Canada in 2020 during growing season.
