# Supplementary material for: Genome-wide association analysis of stress tolerance indices in an interspecific population of chickpea
Source: Front Plant Sci. 2022 Aug 19;13:933277. doi: 10.3389/fpls.2022.933277 (PMC9437449; doi:10.3389/fpls.2022.933277)
Supplement: SUPPLEMENTARY FIGURE 1 — Q–Q plots of the association analysis for seed yield under non-stress (Yp), seed yield under stress conditions (Ys) and yield indices ATI, K1STI, MP, SSPI, and TOL from various GWAS models (CMLM, FarmCPU, FaST-LMM, GLM, MLM, MLMM, SUPER). [file Data_Sheet_1.PDF]

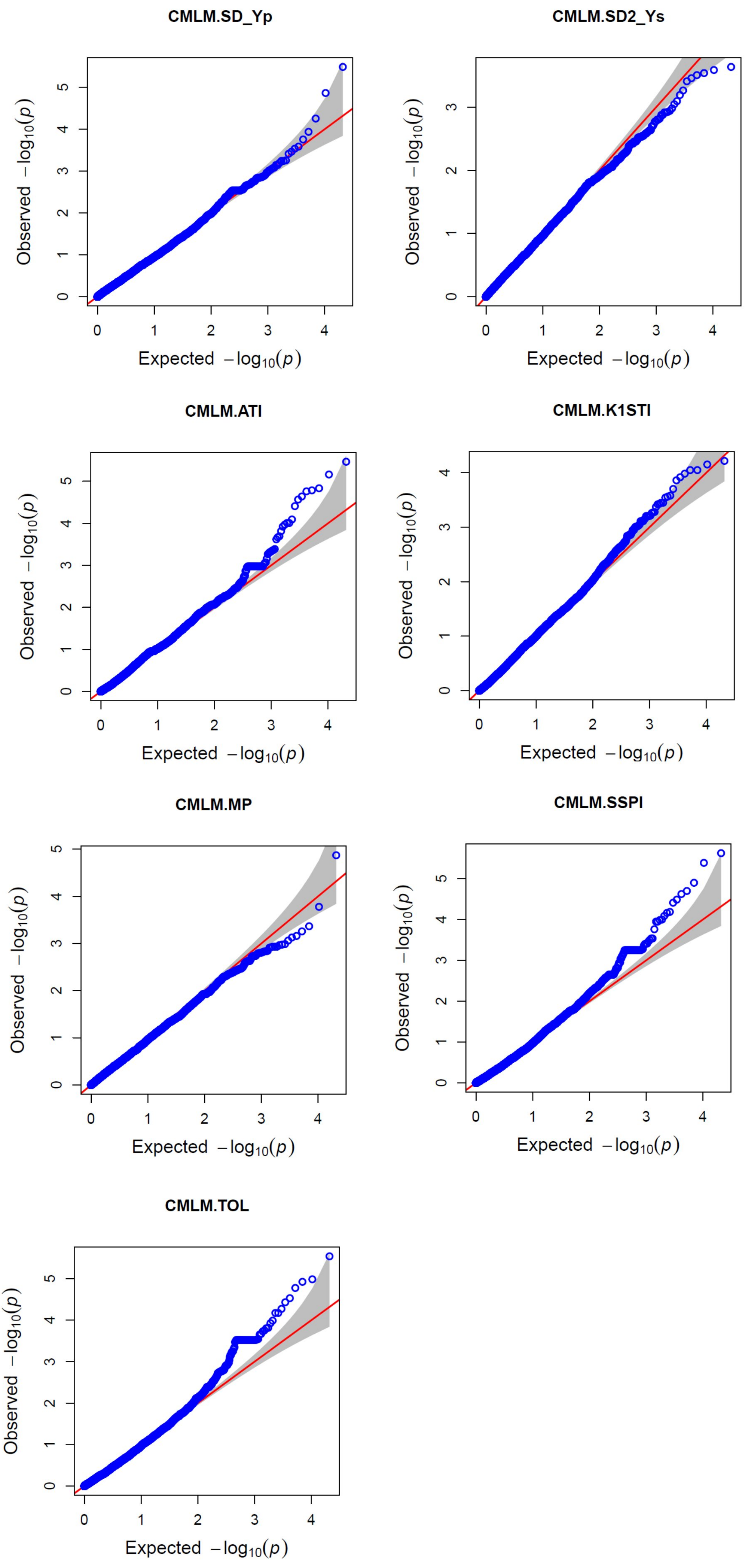
CMLM.SD_Yp
CMLM.SD2_Ys
CMLM.ATI
CMLM.K1STI
CMLM.MP
CMLM.SSPI
CMLM.TOL
Observed $-\log_{10}(p)$
Expected $-\log_{10}(p)$

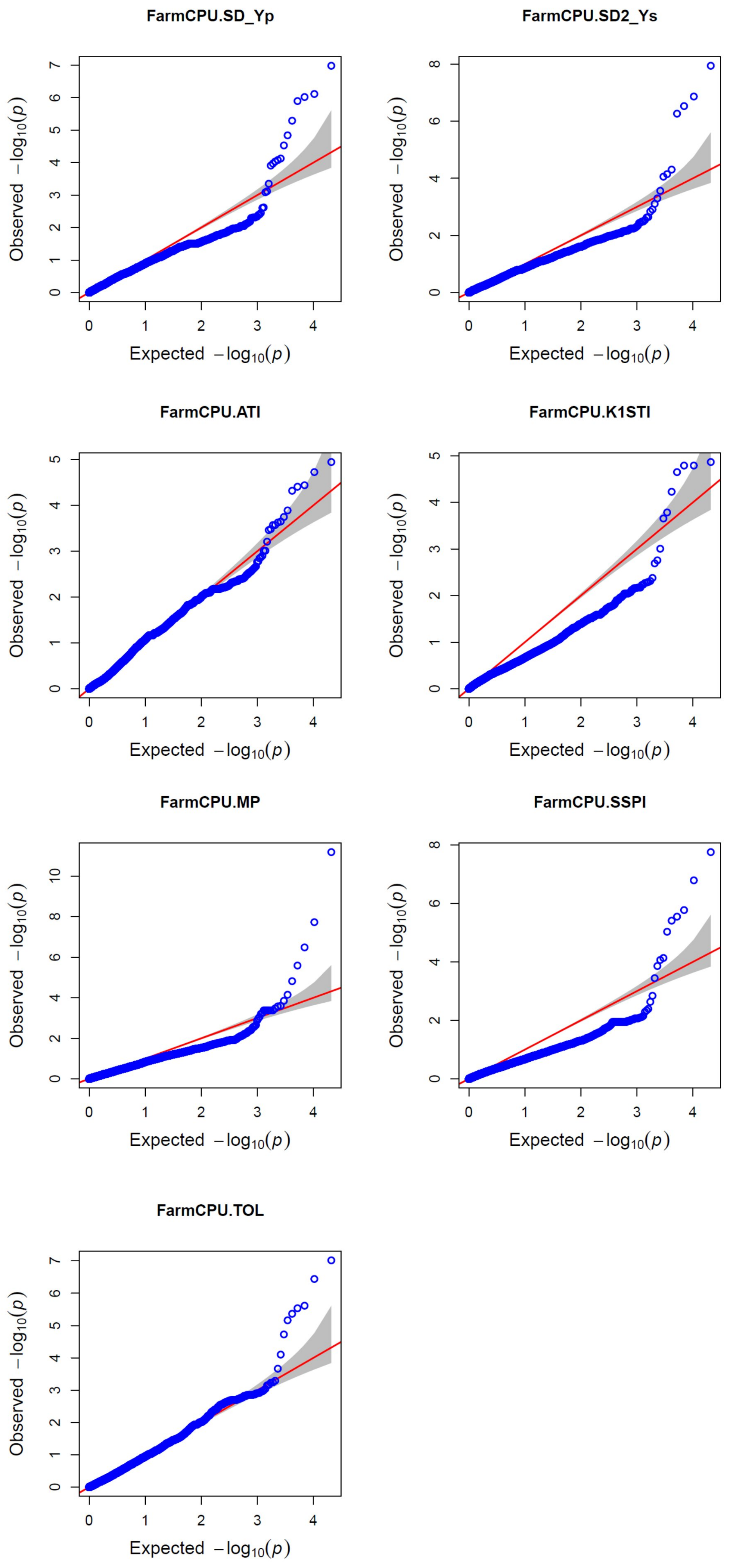
FarmCPU.SD_Yp
FarmCPU.SD2_Ys
FarmCPU.ATI
FarmCPU.K1STI
FarmCPU.MP
FarmCPU.SSPI
FarmCPU.TOL
Observed $-\log_{10}(p)$
Expected $-\log_{10}(p)$

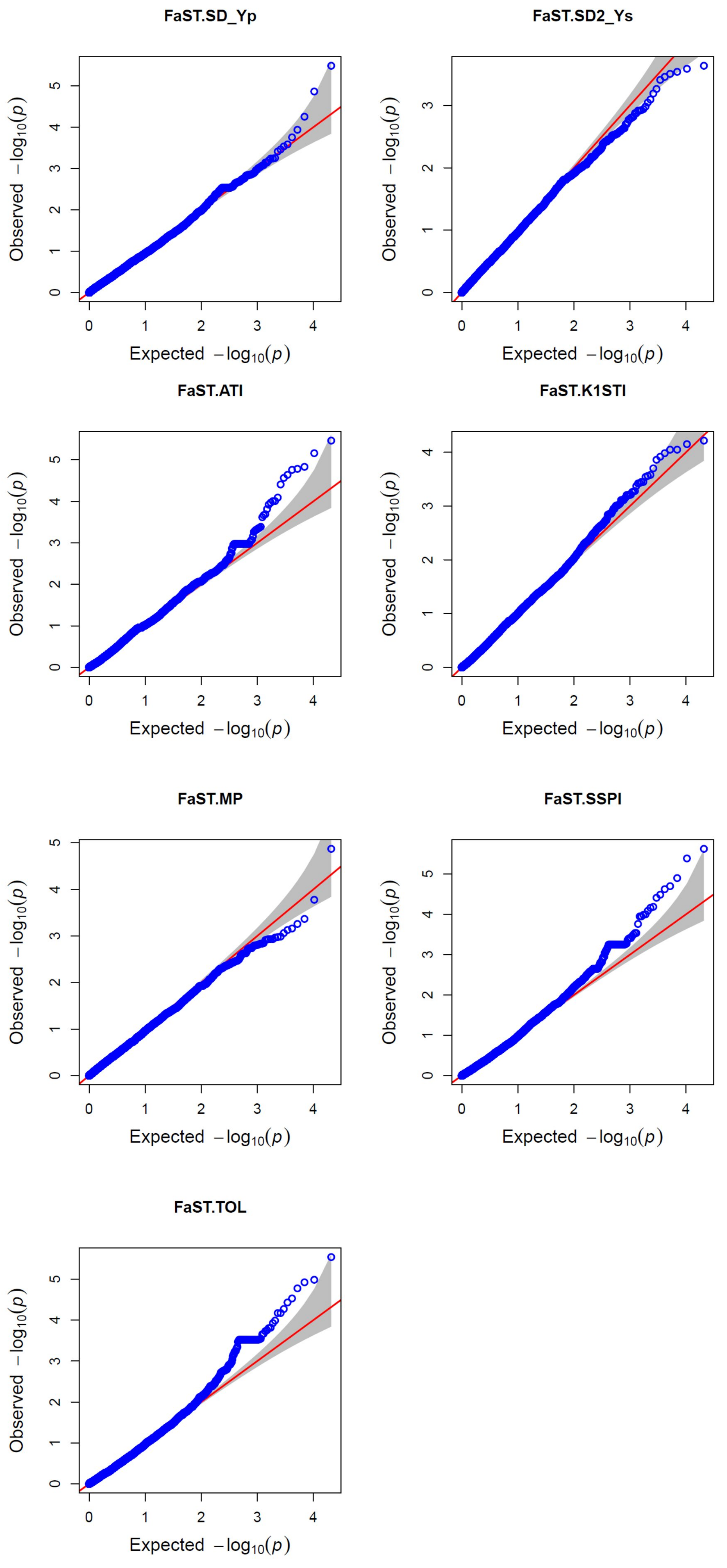
FaST.SD_Yp
FaST.SD2_Ys
FaST.ATI
FaST.K1STI
FaST.MP
FaST.SSPI
FaST.TOL
Observed −log10(p)
Expected −log10(p)

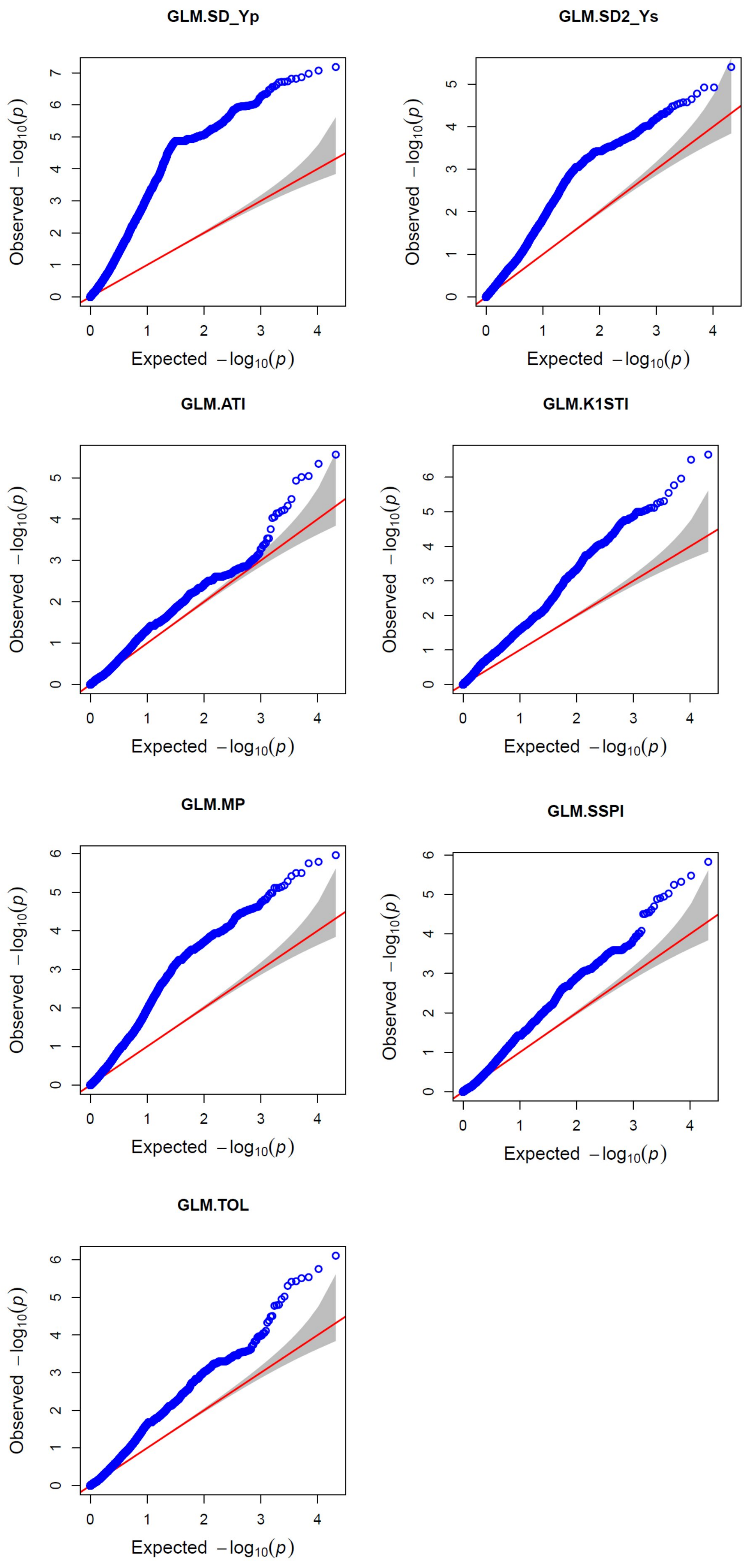
GLM.SD_Yp
GLM.SD2_Ys
GLM.ATI
GLM.K1STI
GLM.MP
GLM.SSPI
GLM.TOL
Observed $-\log_{10}(p)$
Expected $-\log_{10}(p)$

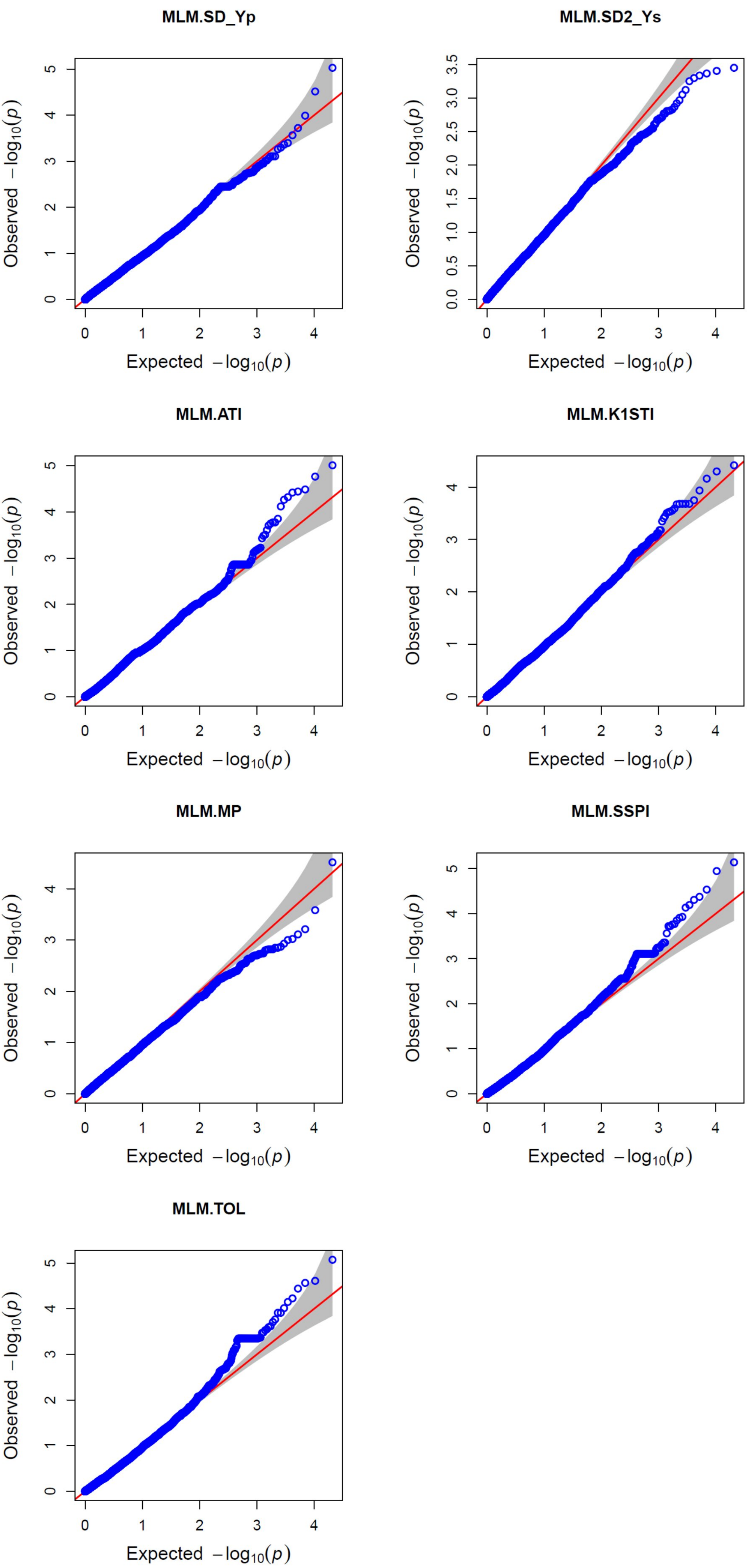

MLM.SD_Yp
MLM.SD2_Ys
MLM.ATI
MLM.K1STI
MLM.MP
MLM.SSPI
MLM.TOL
Observed $-\log_{10}(p)$
Expected $-\log_{10}(p)$

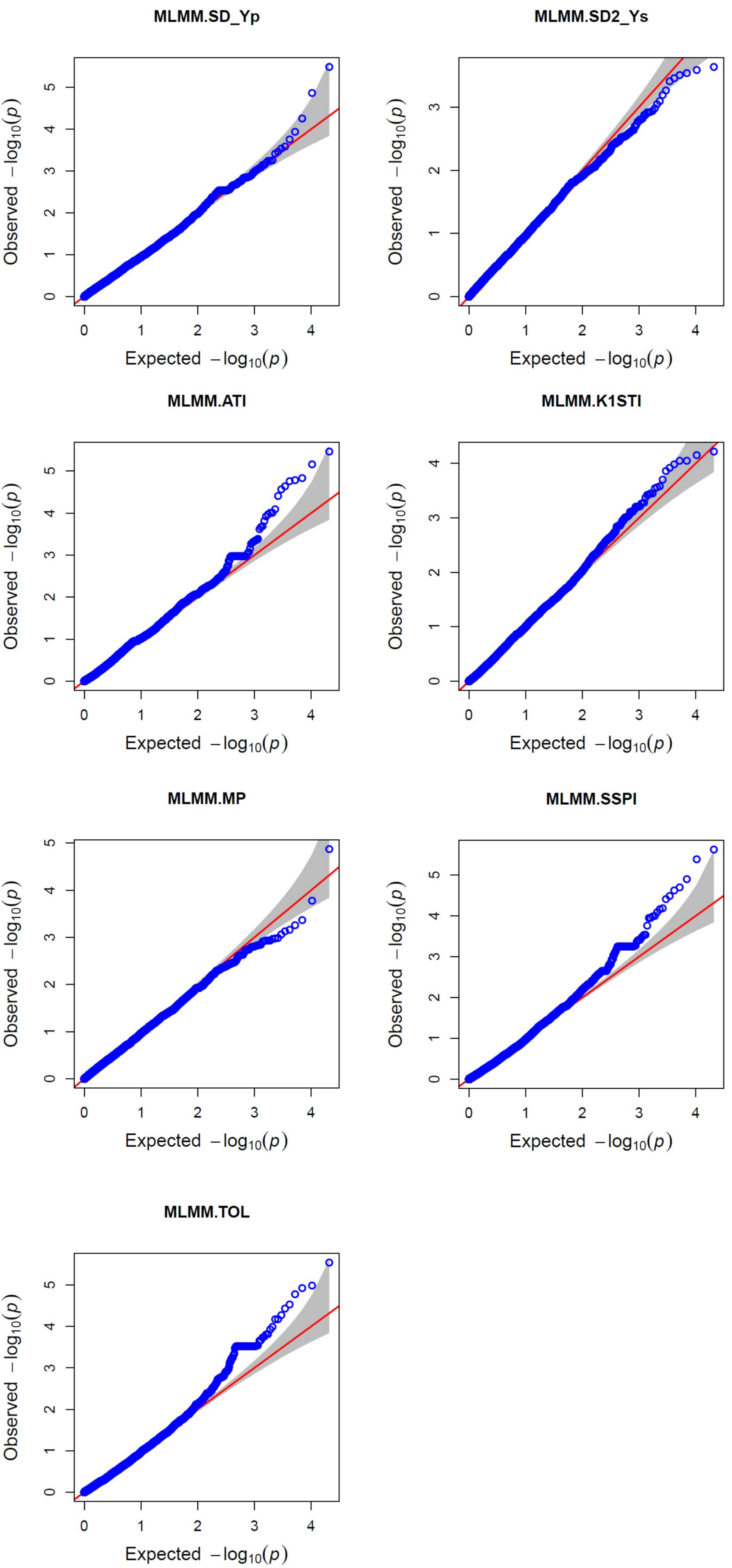
MLMM.SD_Yp
MLMM.SD2_Ys
MLMM.ATI
MLMM.K1STI
MLMM.MP
MLMM.SSPI
MLMM.TOL
Observed $-\log_{10}(p)$
Expected $-\log_{10}(p)$

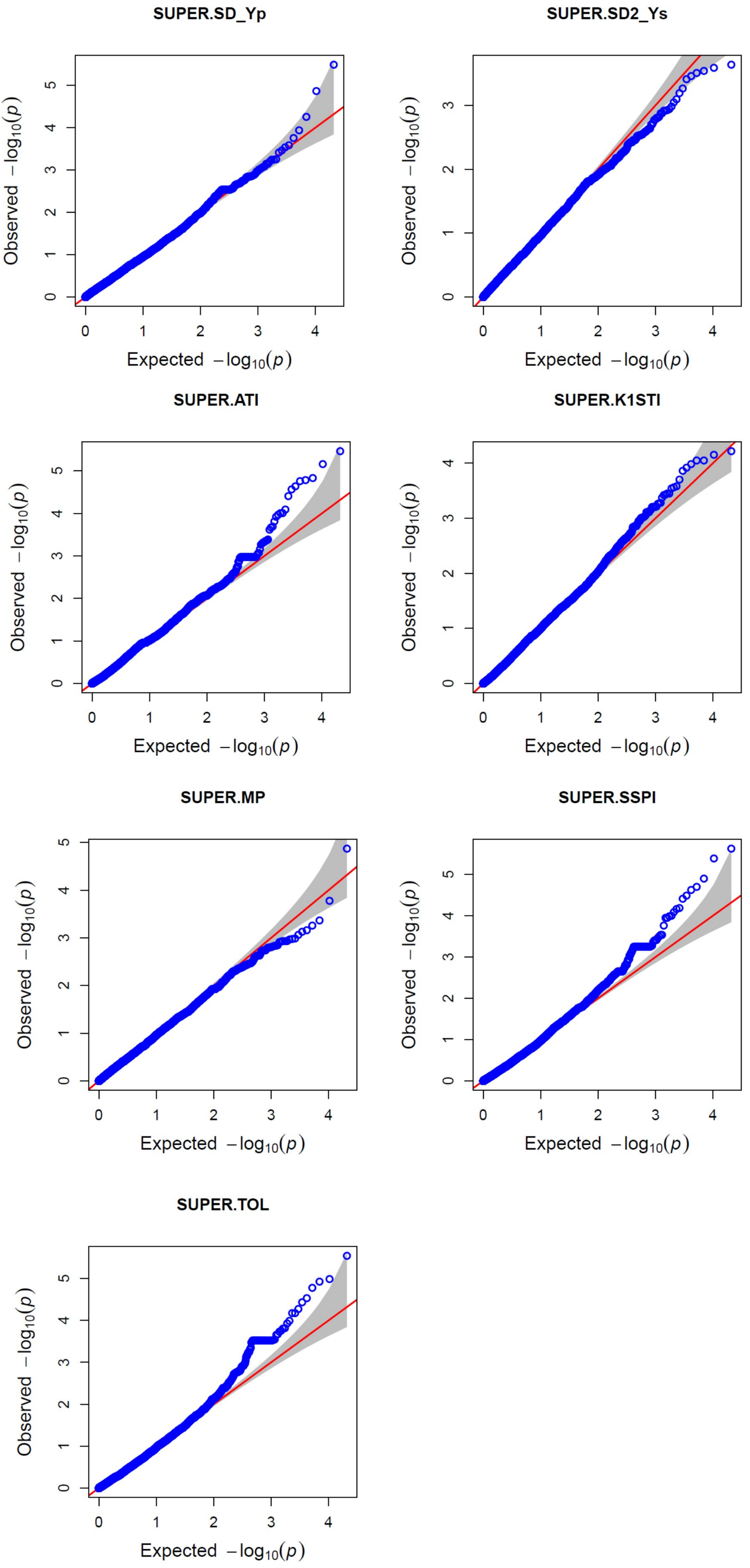
SUPER.SD_Yp
SUPER.SD2_Ys
SUPER.ATI
SUPER.K1STI
SUPER.MP
SUPER.SSPI
SUPER.TOL
Observed $-\log_{10}(p)$
Expected $-\log_{10}(p)$
